# Comparison of effectiveness and safety of cefoperazone–sulbactam versus ceftriaxone and flomoxef in acute cholangitis: a multicenter retrospective study

**DOI:** 10.1128/aac.01829-25

**Published:** 2026-06-12

**Authors:** Chieh-Chang Chen, Jiann-hwa Chen, Wen-Hsin Huang, Tsung-Lin Hsieh, Li-Wei Chen, Ming-Chang Tsai, Nai-Jen Liu, Chi-Huan Wu, Chen-Shuan Chung, Yi-Chun Chiu, Chih-Ming Liang, Cheng-Kun Wu, Wen-Lun Wang, Ching-Tai Lee, Ming-Hung Hsu, Hsiu-Po Wang

**Affiliations:** 1 Division of Gastroenterology and Hepatology, Department of Internal Medicine, National Taiwan University Hospital 38006, Taipei, Taiwan; 2 Department of Internal Medicine, College of Medicine, National Taiwan University 33561, Taipei, Taiwan; 3 Division of Gastroenterology and Hepatology, Taipei Tzu Chi Hospital 145204, New Taipei City, Taiwan; 4 School of Medicine, Tzu Chi University, Hualien, Taiwan; 5 Center for Digestive Medicine, Department of Internal Medicine, China Medical University Hospital 38020, Taichung, Taiwan; 6 Department of Gastroenterology and Hepatology, Chang Gung Memorial Hospital and Chang Gung University at Keelung 56081, Keelung, Taiwan; 7 Division of Gastroenterology and Hepatology, Department of Internal Medicine, Chung Shan Medical University Hospital 63276, Taichung, Taiwan; 8 School of Medicine, Chung Shan Medical University, Taichung, Taiwan; 9 Department of Gastroenterology and Hepatology, Linkou Chang Gung Memorial Hospital 38014, Taoyuan, Taiwan; 10 Division of Gastroenterology and Hepatology, Department of Internal Medicine, Far Eastern Memorial Hospital 46608, New Taipei City, Taiwan; 11 Department of Electrical Engineering, Yuan Ze University 34895, Taoyuan, Taiwan; 12 Division of Hepato-Gastroenterology, Department of Internal Medicine, Kaohsiung Chang Gung Memorial Hospital and Chang Gung University College of Medicine 71589, Kaohsiung, Taiwan; 13 Division of Gastroenterology and Hepatology, Department of Internal Medicine, E-Da Hospital, I-Shou University 54791, Kaohsiung, Taiwan; 14 School of Medicine, College of Medicine, I-Shou University 54791, Kaohsiung, Taiwan; University of Fribourg, Fribourg, Switzerland

**Keywords:** acute cholangitis, cefoperazone–sulbactam, flomoxef, ceftriaxone, empirical antibiotics, biliary tract infection, extended-spectrum β-lactamase (ESBL)

## Abstract

Acute cholangitis is a potentially life-threatening biliary tract infection requiring prompt antibiotic therapy. Cefoperazone–sulbactam is a recommended option for mild-to-moderate disease, but comparative evidence versus ceftriaxone and flomoxef is limited. In this multicenter retrospective study, 389 adults with acute cholangitis from nine Taiwanese centers (January 2022–July 2024) received cefoperazone–sulbactam (*n* = 249), ceftriaxone (*n* = 37), or flomoxef (*n* = 103). The primary outcome was clinical response (cure or improvement) on days 4, 7, and 14. Secondary outcomes were microbiological response, in-hospital mortality, and adverse events (AEs). Multivariable logistic regression was performed, adjusting for age, sex, biliary stenting, comorbidities, malignancy, disease severity, and timing of biliary drainage. Baseline characteristics were similar across groups (mean age, 67 years; 91.8% had mild-to-moderate disease; 33.2% had bacteremia). Day 4 clinical response rates were 96.8% with cefoperazone–sulbactam, 97.3% with ceftriaxone, and 93.2% with flomoxef (*P* = 0.05). Day 7 response did not differ significantly. Compared with cefoperazone–sulbactam, the adjusted odds ratio for day 4 clinical response was 1.40 (95% confidence interval [CI]: 0.17–11.78) for ceftriaxone and 0.50 (95% CI: 0.18–1.43) for flomoxef. AEs were uncommon (2%–5%), mostly mild and not associated with sequelae. Cefoperazone–sulbactam appears to be an effective and safe empiric therapy for acute cholangitis in real-world practice, with high observed early clinical response rates. Although outcomes were statistically similar to those of ceftriaxone and flomoxef, the underpowered between-group comparisons preclude definitive conclusions regarding comparative efficacy. These findings support its continued clinical use while awaiting confirmation in larger randomized trials.

## INTRODUCTION

Acute cholangitis is a severe inflammatory condition of the biliary tract precipitated by bile duct obstruction. It presents as a medical emergency owing to its rapid progression to sepsis and consequent risk of multiorgan failure. Cholangitis treatment requires multidisciplinary management, including hemodynamic resuscitation, antibiotic treatment, and timely biliary drainage (1). Prompt initiation of empiric antibiotic therapy is critical. The Tokyo and major North American and European societies’ guidelines generally recommend third-generation cephalosporins for community-acquired and mild-to-moderate disease (2, 3). However, the effectiveness of antimicrobial therapy varies based on local susceptibility patterns. Furthermore, rising antimicrobial resistance towards enteric pathogens threatens clinical outcomes (4, 5). In the Asia-Pacific region, extended-spectrum β-lactamase (ESBL) production in *Escherichia coli* and *Klebsiella pneumoniae* ranges from 10% to 45% across both community and hospital isolates (6–8). Cefoperazone–sulbactam is a broad-spectrum β-lactam/β-lactamase inhibitor combination drug. It is effective against common enteric pathogens and certain multidrug-resistant organisms, including ESBL-producing *E. coli* and *K. pneumoniae* (9, 10). The Tokyo Guidelines recommend cefoperazone–sulbactam as the first-line antibiotic option for mild-to-moderate acute cholangitis. Nevertheless, clinical evidence on the effectiveness of cefoperazone–sulbactam against standard cephalosporins in cholangitis is limited. In particular, there is a lack of information on early clinical response rates and safety profile in patients with hepatobiliary dysfunction. We therefore compared cefoperazone–sulbactam with ceftriaxone or flomoxef to evaluate the relative effectiveness and safety of these drugs for the treatment of acute cholangitis.

## RESULTS

### Study population

During the initial screening of the study population, 5 patients were excluded specifically due to a baseline international normalized ratio (INR) of >1.5. In total, the study included 389 patients with acute cholangitis (Table 1). Of these, 249 (64.0%) were administered cefoperazone–sulbactam, 37 (9.5%) ceftriaxone, and 103 (26.5%) flomoxef. The mean age of patients across the three groups was approximately 65 years, with no significant between-group differences. The proportion of male patients was similar to that of female patients (53.8% versus 46.2%), with no significant differences between the groups. Most patients (372, 95.6%) had community-acquired infections. Cholelithiasis (*n* = 315, 80.8%) was the leading cause for acute cholangitis in this study population, followed by neoplasm (*n* = 55, 14.1%). Most patients had mild-to-moderate disease (*n* = 357, 91.8%). Diabetes mellitus (*n* = 101, 26.0%), malignancy (*n* = 83, 21.3%), and chronic kidney disease (*n* = 17, 4.4%) were the most prevalent comorbidities, and the mean Charlson Comorbidity Index was 4.1 for all patients. Bacteremia occurred in 129 (33.2%) patients. The most common bloodstream pathogens were *Escherichia coli* (*n* = 80, 72.1%), *Klebsiella* spp. (*n* = 32, 26.4%), *Enterococcus* spp. (*n* = 6, 6.2%), and *Streptococcus* spp. (*n* = 6, 1.3%). Overall, the baseline characteristics were similar between the three groups, including age, sex, etiology, disease severity, comorbidities, and pathogens.

**TABLE 1:** Baseline characteristics

	Cefoperazone–sulbactam (*n* = 249) *n* (%)	Ceftriaxone (*n* = 37) *n* (%)	Flomoxef (*n* = 103) *n* (%)	*P* value
Sex				
Male	141 (56.6)	21 (56.8)	48 (46.6)	0.219
Age, mean (SD)	69.49 (15.41)	67.97 (15.02)	65.83 (15.28)	0.127
Clinical diagnosis				
Cholelithiasis	197 (79.1)	29 (78.4)	89 (86.4)	
Benign stricture	12 (4.8)	0 (0.0)	2 (1.9)	
Bilioenteric anastomotic stricture	2 (0.8)	2 (5.4%)	1 (1.0%)	
Malignant stricture				
Cholangiocarcinoma	8 (3.2)	2 (5.4)	2 (1.9)	
Pancreatic cancer	16 (6.4)	1 (2.7)	7 (6.8)	
Ampulla cancer	2 (0.8)	1 (2.7)	0 (0.0)	
Other malignancy or neoplasm	12 (4.8)	2 (5.4)	2 (1.9)	
Community acquired	234 (94.0)	37 (100)	101 (98)	0.129
Severity grading				0.356
Grade I	142 (57.0)	23 (62.2)	71 (68.9)	
Grade II	85 (34.1)	11 (29.7)	25 (24.3)	
Grade III	22 (8.8)	3 (8.1)	7 (6.8)	
With biliary stent	49 (19.7)	6 (16.2)	30 (29.1)	0.12
Comorbidities				
Diabetes mellitus	66 (26.5)	10 (27.0)	25 (24.3)	0.905
Heart failure	11 (4.4)	2 (5.4)	4 (3.9)	0.860
COPD	10 (4.0%)	1 (2.7)	4 (3.9)	0.999
Chronic liver disease	30 (12.0)	8 (21.6)	12 (11.7)	0.258
Chronic kidney disease	12 (4.8)	1 (2.7)	4 (3.9)	0.999
Malignancy	53 (21.3)	11 (29.7)	19 (18.4)	0.350
Charlson score, mean (SD)	4.3 (2.8)	4.2 (2.3)	3.6 (2.7)	0.143
Bacteremia	88 (35.3)	14 (37.8)	27 (26.2)	0.209
*E. coli*	58	7	15	
*Klebsiella* spp.	17	5	10	
*Enterococcus* spp.	6	1	1	
*Streptococcus* spp.	5	0	1	
*Enterobacter* spp.	1	0	1	
Others	12	5	4	

### Clinical efficacy

Detailed results of antibiotic treatment and biliary drainage procedures are summarized in Table 2. High day 4 clinical response rates (cure plus improvement) were observed across all three groups, including cefoperazone–sulbactam (96.8%), ceftriaxone (97.3%), and flomoxef (93.2%) groups (overall *P* = 0.050). A detailed categorical breakdown of specific early clinical outcomes (clinical cure versus clinical improvement) across all evaluated time points is provided in Table S1. Furthermore, the early kinetic trajectories of objective clinical parameters—including hemodynamic stabilization and the clearance of systemic inflammatory markers from baseline to day 4—were statistically similar among all three regimens (Table S2). There were no significant between-group differences in clinical response at day 7. Similarly, microbiological outcomes at days 4 and 7 did not differ significantly among the cohorts. The in-hospital mortality rate was similar and remarkably low across the cefoperazone–sulbactam (0.8%), ceftriaxone (0%), and flomoxef (1.0%) groups (*P* = 0.99). The mean length of hospital stay was also comparable (9.7, 10.1, and 8.3 days, respectively; *P* = 0.136), with no significant differences observed in the rates of ICU admission or new-onset organ failure.

**TABLE 2:** Treatment outcomes

	Cefoperazone–sulbactam (*n* = 249) *n* (%)	Ceftriaxone (*n* = 37) *n* (%)	Flomoxef (*n* = 103) *n* (%)	*P* value
Hospital stay (day), mean (SD)	9.73 (6.86)	10.05 (6.54)	8.29 (5.60)	0.136
ICU	1 (0.4)	0 (0.0)	1 (1.0)	0.591
Death	2 (0.8)	0 (0.0)	1 (1.0)	0.999
New-onset organ failure	7 (2.8)	0 (0.0)	1 (1.0)	0.531
Biliary drainage				0.230
None	42 (16.9)	5 (13.5)	14 (13.6)	
Failure	5 (2)	2 (5.4)	1 (1.0)	
Endoscopic	178 (71.5)	29 (78.4)	82 (79.6)	
Radiological	22 (8.8)	0 (0.0)	6 (5.8)	
Surgical	2 (0.8)	1 (2.7)	0 (0.0)	
Timing of drainage**^*a*^**	*n* = 203	*n* = 30	*n* = 88	0.253
≤24 h	86 (42.4)	12 (40.0)	46 (52.3)	
>24 h	117 (57.6)	18 (60.0)	42 (47.7)	
Clinical outcome				
Day 4 (*n* = 388)				
Clinical response	240 (96.8)	36 (97.3)	96 (93.2)	0.05
Indeterminate	6 (2.4)	0 (0.0)	2 (1.9)	
Failure	2 (0.8)	1 (2.7)	5 (4.9)	
Day 7 (*n* = 312)				0.50
Clinical response	205 (97.2)	25 (100.0)	74 (97.4)	
Indeterminate	0 (0.0)	0 (0.0)	1 (1.3)	
Failure	6 (2.8)	0 (0.0)	1 (1.3)	
Day 14 (*n* = 208)				0.19
Clinical response	139 (98.6)	16 (94.1)	50 (100)	
Indeterminate	0 (0.0)	0 (0.0)	0 (0.0)	
Failure	2 (1.4)	1 (5.9)	0 (0.0)	
Microbiological outcome				
Day 4 (*n* = 388)				
Microbiological response	224 (90.3)	34 (91.9)	95 (92.2)	0.085
Indeterminate	9 (3.6)	3 (8.1)	3 (2.9)	
Persistence	15 (6.0)	0 (0.0)	5 (4.9)	
Day 77 (*n* = 265)				
Microbiological response	175 (96.2)	23 (100.0)	60 (100.0)	0.831
Indeterminate	4 (2.2)	0 (0.0)	0 (0.0)	
Persistence	3 (1.6)	0 (0.0)	0 (0.0)	

^*a*^ Timing of drainage is calculated based on patients with successful biliary decompression.

In multivariable logistic regression analysis (Table 3), older age, sex, disease severity, malignant stricture, biliary stent use, timing of biliary drainage, and comorbidities were not associated with day 4 clinical response. The odds of achieving clinical response did not differ significantly for ceftriaxone (odds ratio [OR]: 1.40, 95% confidence interval [CI]: 0.17–11.78), or flomoxef (OR: 0.50, 95% CI: 0.18–1.43) relative to cefoperazone–sulbactam.

**TABLE 3:** Multivariable analysis for early clinical response

	Odds ratio	*P* value	95% CIs
Age ≥65 years	0.81	0.75	0.22–2.92
Male	0.72	0.53	0.26–1.99
Severity			
Grade 1	1.00 (reference)		
Grade 2	0.79	0.70	0.24–2.62
Grade 3	0.29	0.12	0.06–1.36
Charlson score	1.08	0.52	0.85–1.39
Malignant stricture	1.29	0.65	0.43–3.84
Prior biliary stenting	0.52	0.24	0.18–1.53
Biliary drainage timing			
Drainage ≤24 h	1.00 (reference)		
No drainage/failure	0.46	0.34	0.10–2.26
Drainage >24 h	0.43	0.17	0.13–1.44
Treatment			
Cefoperzone–sulbactam	1.00 (reference)		
Ceftriaxone	1.40	0.76	0.17–11.78
Flomoxef	0.50	0.20	0.18–1.43

### Adverse effects

Overall, all three antibiotics were well tolerated in patients with acute cholangitis. Adverse-event rates are summarized in Table 4. The few reported adverse events included diarrhea (*n* = 9, 3.6% with cefoperazone–sulbactam), eosinophilia (*n* = 8, 3.2% with cefoperazone–sulbactam; *n* = 4, 3.9% with flomoxef), and skin eruption (*n* = 1, 2.7% with ceftriaxone). In the cefoperazone–sulbactam group, 69 patients (27.7%) received prophylactic vitamin K infusion once a week. Prolonged prothrombin time (PT) was observed in 6 patients (2.4%) in the cefoperazone–sulbactam group and in 1 patient (1%) in the flomoxef group. No adverse event resulted in short- or long-term sequelae.

**TABLE 4:** Adverse events

	Cefoperazone–sulbactam (*n* = 249) *n* (%)	Ceftriaxone (*n* = 37) *n* (%)	Flomoxef (*n* = 103) *n* (%)
Hepatobiliary			
ALP↑, GGT↑	5 (2)	0 (0)	1 (1)
AST↑, GGT↑	6 (2.4)	0 (0)	3 (2.9)
Jaundice	0 (0)	1 (2.7)	0
Hematology			
Anemia	6 (2.4)	0 (0)	6 (5.8)
Leukopenia	0 (0)	0 (0)	1 (1.0)
Thrombocytopenia	0 (0)	0 (0)	1 (1.0)
Eosinophilia	8 (3.2)	0 (0)	4 (3.9)
Neutropenia	1 (0.4)	0 (0)	1 (1.0)
Prolonged PT, PTT	6 (2.4)	0 (0)	1 (1.0)
Gastroenterology			
Constipation	1 (1.0)	0 (0)	1 (1.0)
Diarrhea	9 (3.6)	0 (0)	0 (0)
Nausea	0 (0)	1 (2.7)	0 (0)
Epigastric pain	1 (0.4)	0 (0)	0 (0)
Gastrointestinal bleeding	1 (0.4)	0 (0)	2 (1.9)
Phlebitis	0 (0)	0 (0)	0 (0)
Skin rash	0 (0)	1 (2.7)	0 (0)

## DISCUSSION

To our knowledge, this is the largest study to evaluate cefoperazone–sulbactam as empiric therapy for acute cholangitis. In this multicenter real-world cohort, cefoperazone–sulbactam was associated with high clinical response rates and a favorable safety profile, with outcomes broadly comparable to those of ceftriaxone and flomoxef. Although the unadjusted day 4 response rates for cefoperazone–sulbactam and ceftriaxone (96.8% and 97.3%) were marginally higher than for flomoxef (93.2%), antibiotic choice was not independently associated with early response after multivariable adjustment.

The high clinical efficacy observed across all regimens is in accordance with the Tokyo Guidelines 2018 recommendations (2). The present results are consistent with prior reports showing that cefoperazone–sulbactam is effective in managing acute cholangitis (11–16). Gao et al., Jiao et al., and Wang et al. reported superior or comparable outcomes for cefoperazone–sulbactam compared with other agents (11, 13, 14). A recent systematic review and meta-analysis further confirmed similar clinical efficacy and safety between cefoperazone–sulbactam and comparator antibiotics in intra-abdominal infections (16). Although they reported that ceftriaxone was associated with faster symptom relief and bacterial clearance by day 3 (72.0% versus 41.3%), overall effectiveness was highly comparable between the groups (~98%) (12). These variations in early response rates across studies are likely attributable to differences in patient populations, accompanying interventions, and the lack of standardized efficacy criteria. In particular, clinical success was defined differently across studies, including normalization of inflammatory and hepatic laboratory markers in some reports (11, 13, 14) and resolution of clinical symptoms such as fever in others (12).

Current guidelines recommend tailoring empiric therapy to disease severity, local resistance patterns, and patient-specific risk factors (2, 3). Empiric treatment regimens should cover common pathogens, including Enterobacterales and *Enterococcus* spp., and address potentially resistant organisms such as ESBL-producing Enterobacterales and *Pseudomonas aeruginosa*. Third-generation cephalosporins are generally suggested for mild or community-acquired infections. However, rising antimicrobial resistance in Enterobacterales isolated from cholangitis has been reported (7, 17, 18), which may contribute to treatment failure and worse outcomes (18, 19). Some of the risk factors recognized for resistance are indwelling biliary devices, nosocomial acquisition, prior biliary interventions, and recent antibiotic exposure (5, 7). In our cohort, approximately 40% had moderate-to-severe disease, and 22% had biliary stents. Because biliary devices promote biofilm formation and are associated with prior healthcare exposure, they may increase the risk of infection due to resistant organisms, underscoring the importance of selecting agents with reliable activity in higher-risk clinical settings. Cefoperazone–sulbactam provides broad-spectrum activity against anaerobes and various gram-negative organisms, including ESBL-producing and non-ESBL-producing Enterobacterales, *P. aeruginosa*, and other non-fermenting bacilli (10, 20). This microbiologic profile may partly explain the high observed early response rate in the cefoperazone–sulbactam group. However, the between-group differences were not statistically significant after multivariable adjustment, likely reflecting limited power to detect modest treatment effects.

Safety is an important consideration in antimicrobial therapy. Across the three groups, adverse event rates were low (~2% to 5%), and all regimens were generally well tolerated. Both cefoperazone–sulbactam and flomoxef can interfere with vitamin K-dependent coagulation through the N-methylthiotetrazole side chain (21, 22). Although this complication is uncommon, it may be more relevant in patients with malnutrition, prior hemorrhagic events, or concomitant anticoagulants (21). In our study, prolonged PT was observed in 6 patients (2.4%) administered cefoperazone–sulbactam and in 1 patient (1.0%) administered flomoxef. Notably, no major bleeding events or adverse outcomes leading to sequelae or death were observed. Collectively, these data support an acceptable safety profile for cefoperazone–sulbactam in acute cholangitis when appropriate monitoring is applied.

This study has several limitations. First, its retrospective, non-randomized design inherently introduces the possibility of confounding by indication and channeling bias. Physicians may have preferentially selected broader-spectrum agents such as cefoperazone–sulbactam for patients perceived to be at higher risk of resistant pathogens or more severe clinical courses. Although we attempted to mitigate this by incorporating critical risk factors, such as prior biliary stents and timing of biliary drainage, into our multivariable regression model, residual confounding from unmeasured variables (e.g., prior antibiotic exposure, prior colonization with resistant organisms, or local resistance patterns) may still persist. Second, the unequal group sizes (cefoperazone–sulbactam, *n* = 249; ceftriaxone, *n* = 37; flomoxef, *n* = 103) likely reflect these center- and prescriber-level treatment preferences. In particular, the small ceftriaxone sample limits statistical power and contributes to wide confidence intervals (CIs) in between-group comparisons. Nevertheless, the multicenter design enhances generalizability, and the concordant results across multiple endpoints support the overall robustness of the findings. Prospective, randomized trials are ultimately needed to eliminate this bias and confirm comparative efficacy. Third, retrospective data collection inherently risks the underreporting of minor adverse events. Furthermore, by excluding patients with a baseline INR of >1.5 (*n* = 5), we removed a population with preexisting coagulopathy, which likely underestimates the true real-world bleeding risk. Consequently, our safety findings cannot be generalized to patients at high baseline bleeding risk. Nevertheless, within our studied cohort, overall adverse-event frequencies remained similar to previous reports (15, 23–25), and most events were mild. Fourth, detailed isolate-level microbiology and susceptibility data—particularly within the bacteremia subgroup—were not uniformly available. Because antimicrobial susceptibility testing protocols varied across the nine participating institutions, routine testing and reporting for specific agents like flomoxef and cefoperazone–sulbactam were frequently omitted. Consequently, a comprehensive susceptibility profile for key pathogens (such as *Escherichia coli* and *Klebsiella* spp*.*) could not be provided to correlate directly with clinical effectiveness.

In summary, cefoperazone–sulbactam appears to be an effective and well-tolerated empiric treatment option for acute cholangitis, with high observed early clinical response rates in this multicenter cohort. Although adjusted analyses did not show statistically significant differences versus ceftriaxone or flomoxef, the limited power of the between-group comparisons precludes definitive conclusions regarding comparative efficacy. Larger randomized controlled trials are warranted to confirm these findings.

## MATERIALS AND METHODS

### Study design and participants

This was a retrospective, multicenter study conducted at nine medical centers in Taiwan. Patients aged ≥20 years and diagnosed with acute cholangitis between January 2022 and July 2024 were included if they had received treatment with cefoperazone–sulbactam (Brosym; TTY Biopharm Company, Taiwan), ceftriaxone, or flomoxef. Patients were excluded if they had *Mycobacterium tuberculosis* infection, disseminated intravascular coagulation, Child–Pugh C liver cirrhosis, an international normalized ratio (INR) of >1.5 times the upper limit of normal, or were administered an antibiotic course <4 days. The diagnosis and severity of acute cholangitis were established based on Tokyo Guidelines 2018 (26).

### Outcome measures

The primary outcome was the proportion of patients who achieved clinical response defined as either clinical cure (complete resolution of baseline signs and symptoms) or clinical improvement (partial resolution of baseline signs and symptoms). This was assessed on days 4, 7, and 14. Clinical failure was defined as a requirement for antibiotic escalation or death attributable to acute cholangitis after ≥3 days of therapy. Outcomes were considered indeterminate if data to determine success or failure of treatment were insufficient. Secondary outcomes were microbiological response, in-hospital mortality (related or unrelated to acute cholangitis), and antibiotic-associated adverse events.

Microbiological responses were assessed on days 4, 7, and 14. Microbiological response was defined as either eradication (≥1 set of negative cultures from the same site) or presumed eradication (culture report indicating mixed flora/contamination, culture not available, or the isolates being different from the original strain, clinical outcomes being interpreted as cure or improvement). An indeterminate outcome was defined as a culture report with insufficient clinical data to classify response. Persistence indicated at least one positive culture from the same site, with the same strain identified as the original isolate. Adverse events were ascertained through manual review of daily progress notes, consultation notes, laboratory results, and discharge summaries, rather than by a predefined keyword-based search. The severity of antibiotic-associated adverse events was graded according to Common Terminology Criteria for Adverse Events (version 5.0).

### Statistical analysis

Data were analyzed using R software (version 4.5.1; R Foundation for Statistical Computing, Vienna, Austria). All continuous variables were reported as means with standard deviations or medians with interquartile ranges. Categorical variables were reported as *n* (%). For univariate analysis, continuous data were compared using one-way ANOVA, and categorical data were compared using *χ*^2^ test or Fisher’s exact test, as appropriate. All univariate comparisons were unpaired, and significance tests were two tailed.

Multivariable logistic regression analyses were used to calculate odds ratios (ORs) and 95% confidence intervals (CIs) for the association between antibiotic group (ceftriaxone or flomoxef versus cefoperazone–sulbactam [reference]) and clinical response, after adjustment for age, sex, comorbidities, malignant stricture, prior biliary stenting, timing of biliary drainage, and disease severity.

## SUPPLEMENTAL MATERIAL

Supplemental material aac.01829-25-s0001.docx Tables S1 and S2.

ASM does not own the copyrights to Supplemental Material that may be linked to, or accessed through, an article. The authors have granted ASM a non-exclusive, world-wide license to publish the Supplemental Material files. Please contact the corresponding author directly for reuse.
