# Supplementary material for: Comparison of effectiveness and safety of cefoperazone–sulbactam versus ceftriaxone and flomoxef in acute cholangitis: a multicenter retrospective study
Source: Antimicrob Agents Chemother. 2026 Jun 12;70(7):e01829-25. doi: 10.1128/aac.01829-25 (PMC13321815; doi:10.1128/aac.01829-25)
Supplement: Supplemental material — Tables S1 and S2. [file aac.01829-25-s0001.docx]

**Supplementary Table S1. Detailed clinical outcomes at Days 4, 7, and 14 Following Treatment with Cefoperazone/sulbactam, Ceftriaxone, or Flomoxef**

|  | Cefoperazone/sulbactam (n=249) | |  | Ceftriaxone (n=37) | |  | Flomoxef (n=103) | |  |
| --- | --- | --- | --- | --- | --- | --- | --- | --- | --- |
|  | n | % |  | n | % |  | n | % | P-value |
| **Clinical outcome** |  |  |  |  |  |  |  |  |  |
| Day4 (n=388) |  |  |  |  |  |  |  |  | 0.050 |
| Cure | 74 | 29.8% |  | 7 | 18.9% |  | 38 | 36.9% |  |
| Improvement | 166 | 66.9% |  | 29 | 78.4% |  | 58 | 56.3% |  |
| Cure+Improvement | 240 | 96.8% |  | 36 | 97.3% |  | 96 | 93.2% |  |
| Indeterminate | 6 | 2.4% |  | 0 | 0.0% |  | 2 | 1.9% |  |
| Failure | 2 | 0.8% |  | 1 | 2.7% |  | 5 | 4.9% |  |
| Indeterminate+Failure | 8 | 3.2% |  | 1 | 2.7% |  | 7 | 6.8% |  |
| Day7 (n=312) |  |  |  |  |  |  |  |  | 0.504 |
| Cure | 132 | 62.6% |  | 13 | 52.0% |  | 47 | 61.8% |  |
| Improvement | 73 | 34.6% |  | 12 | 48.0% |  | 27 | 35.5% |  |
| Cure+Improvement | 205 | 97.2% |  | 25 | 100.0% |  | 74 | 97.4% |  |
| Indeterminate | 0 | 0.0% |  | 0 | 0.0% |  | 1 | 1.3% |  |
| Failure | 6 | 2.8% |  | 0 | 0.0% |  | 1 | 1.3% |  |
| Indeterminate+Failure | 6 | 2.8% |  | 0 | 0.0% |  | 2 | 2.6% |  |
| Day14 (n=208) |  |  |  |  |  |  |  |  | 0.194 |
| Cure | 116 | 82.3% |  | 15 | 88.2% |  | 38 | 76.0% |  |
| Improvement | 23 | 16.3% |  | 1 | 5.9% |  | 12 | 24.0% |  |
| Cure+Improvement | 139 | 98.6% |  | 16 | 94.1% |  | 50 | 100.0% |  |
| Indeterminate | 0 | 0.0% |  | 0 | 0.0% |  | 0 | 0.0% |  |
| Failure | 2 | 1.4% |  | 1 | 5.9% |  | 0 | 0.0% |  |
| Indeterminate+Failure | 2 | 1.4% |  | 1 | 5.9% |  | 0 | 0.0% |  |

**Supplementary Table S2. Changes in clinical parameters and laboratory markers from baseline to day 4 during treatment with Cefoperazone-sulbactam, Ceftriaxone, or Flomoxef**

|  | **Cefoperazone-sulbactam** | **Ceftriaxone** | **Flomoxef** | *p* value |
| --- | --- | --- | --- | --- |
| Defervescence, days | 1.71 (8.68), N=115 | 3.4 (2.82), N=15 | 2.79 (1.35), N=42 | 0.55 |
| MAP, mmHg |  |  |  |  |
| D1 | 90 (80 ~ 100) | 92.8 (82.3 ~ 103.3) | 92.8 (82 ~ 102.6) | 0.70 |
| D4 | 94.05 (84.85 ~ 101.55) | 91.2 (86.6 ~ 99.9) | 93.15 (86.3 ~101.3) | 0.86 |
| Absolute change from D1 | 2.9 (-7.45 ~ 13.35) | -1 (-10.6 ~ 9.70) | 0.35 (-10.2 ~ 11) | 0.19 |
| Pulse rate, |  |  |  |  |
| D1 | 85 (76 ~ 98) | 80 (69 ~ 98) | 82.5 (72 ~ 98) | 0.24 |
| D4 | 74 (66 ~ 84) | 76 (67 ~ 86) | 74 (65 ~ 81) | 0.38 |
| Absolute change from D1 | -10 (0 ~ -20) | -4 (3 ~ -15) | -10 (3 ~ -23) | 0.27 |
| WBC count, 10^3^/uL |  |  |  |  |
| D1 | 10.1 (7.4 ~ 13.6) | 10.7 (7.2 ~ 14.2) | 9.6 (6.8 ~ 12.3) | 0.41 |
| D4 | 6.4 (5.2 ~ 8.2) | 7.7 (5.6 ~ 9.0) | 6.0 (4.8 ~ 7.8) | 0.08 |
| Decrease from D1 (%) | 34.93 (12.86 ~ 12.86) | 26.17 (5.88 ~ 49.15) | 33.90 (9.27 ~ 50) | 0.63 |
| Bil-T, mg/dL |  |  |  |  |
| D1 | 3.38 (1.98 ~ 5.6) | 3.4 (1.7 ~ 5.1) | 3.22 (2.1 ~ 5.56) | 0.93 |
| D4 | 1.4 (0.88 ~ 2.74) | 1.56 (0.9 ~ 2.8) | 1.6 (1.01 ~ 2.65) | 0.63 |
| Decrease from D1 (%) | 51.28 (51.28 ~ 66.67) | 42.70 (10.0 ~ 65.64) | 48.64 (20.12 ~ 67.74) | 0.76 |
| C-reactive protein, mg/dL | N = 144 | N=22 | N=68 |  |
| D1 | 4.63 (2.22 ~ 8.73) | 2.75 (1.51 ~ 6.53) | 4.00 (1.09 ~ 8.54) | 0.29 |
| D4 | 2.81 (1.21 ~ 6.5) | 2.5 (1.59 ~ 3.08) | 1.65 (0.85 ~ 3.47) | 0.14 |
| Decrease from D1 (%) | 41.16 (24.16 ~ 70.49) | 21.50 (14.97 ~ 71.72) | 57.13 (72.57 ~ 22.45) | 0.40 |
